# Co-evolution of wildfires and early *Sphagnum*-peatlands near the Cretaceous South Pole

**DOI:** 10.1038/s43247-026-03959-1

**Published:** 2026-09-07

**Authors:** Ulrich Salzmann, Thorsten Bauersachs, Johann P. Klages, Henny Gerschel, Juliane Müller, Immo Heinz, Claus-Dieter Hillenbrand, Steven M. Bohaty, Torsten Bickert, Jürgen Titschack, Sergej Kaschuro, James McKay, Gernot Nehrke, Heiko Pälike, the Science Team of Expedition PS104, Jan Erik Arndt, Werner Ehrmann, Oliver Esper, Thomas Frederichs, Tim Freudenthal, Catalina Gebhardt, Karsten Gohl, Katharina Hochmuth, Gerhard Kuhn, Robert Larter, Gerrit Lohmann, Thomas Ronge, Patric Simões Pereira, James A. Smith, Cornelia Spiegel, Gabriele Uenzelmann-Neben, Tina van de Flierdt

**Affiliations:** 1 School of Geography and Natural Sciences, Northumbria University, Newcastle upon Tyne, UK; 2 Chair of Organic Biogeochemistry in Geo-Systems, RWTH Aachen University, Aachen, Germany; 3 Alfred-Wegener-Institut Helmholtz-Zentrum für Polar- und Meeresforschung, Bremerhaven, Germany; 4 Cluster of Excellence “The Ocean Floor – Earth’s Uncharted Interface”, University of Bremen, Bremen, Germany; 5 Arbeitsgemeinschaft für Kohle und Organische Petrologie (AKOP) / Arbeitskreis Bergbaufolgen der DGGV e. V., Freiberg, Germany; 6 Organic Petrology and Geochemistry, Institute of Geology, TU Bergakademie Freiberg, Freiberg, Germany; 7 Thünen Institute of Wood Research, Hamburg, Germany; 8 British Antarctic Survey, Cambridge, UK; 9 Institute of Earth Sciences, University of Heidelberg, Heidelberg, Germany; 10 MARUM – Center for Marine Environmental Sciences, University of Bremen, Bremen, Germany; 11 School of Earth, Environment and Sustainability, University Leeds, Leeds, UK; 12 Institute of Geomatics, Boku University, Wien, Austria; 13 Institute for Earth System Science and Remote Sensing, University Leipzig, Leipzig, Germany; 14 Institute for Marine and Antarctic Studies, Oceans and Cryosphere, University of Tasmania, Hobart, TAS Australia; 15 Texas A&M University, College Station, TX USA; 16 Institute of Geology, University of Innsbruck, Innsbruck, Austria; 17 Department of Geosciences, University of Bremen, Bremen, Germany; 18 Department of Earth & Engineering, Imperial College London, London, UK

**Keywords:** Palaeoclimate, Palaeoecology

## Abstract

Peatlands are important global carbon sinks but face increasing fire vulnerability under projected warmer and CO_2_-rich climate conditions. Past high-CO_2_ periods in Earth’s history serve as an important prospect to improve our understanding of fire regime changes and linked ecosystem responses. Here, we report wildfires in Late Cretaceous temperate rainforests and wetlands that existed only ~ 900 km away from the South Pole. Multiple proxy evidence from macro- and microcharcoal, polycyclic aromatic hydrocarbons, maceral analysis, inertinite reflectance and charcoal morphology document frequent, low-temperature surface fires. The fire regime changes closely coincide with a hydroseral succession (i.e., terrestrialisation) from swamp forests to the earliest known coal-forming *Sphagnum*-peat bog from southern high latitudes. Despite fundamental differences in atmospheric and floristic composition, fire and vegetation in 90-million-year-old Cretaceous forests and peatlands appear to have interacted in ways remarkably similar to modern ecosystems, with recurrent burning maintaining open vegetation structure and promoting peatland development. Our deep-time study establishes fire as an intrinsic driver of high-latitude wetland ecosystems and provides critical constraints on fire regime responses and ecosystem resilience under warm, elevated-CO₂ greenhouse conditions.

## Abstract (web summary)

Pollen, charcoal, and biomarkers from the 90-million-year-old peatland sediments in Antarctica reveal that fire dynamics in the earliest peatlands of the warm, CO₂-rich Cretaceous world closely resembled those of modern ecosystems.

## Introduction

**Fig. 1:**
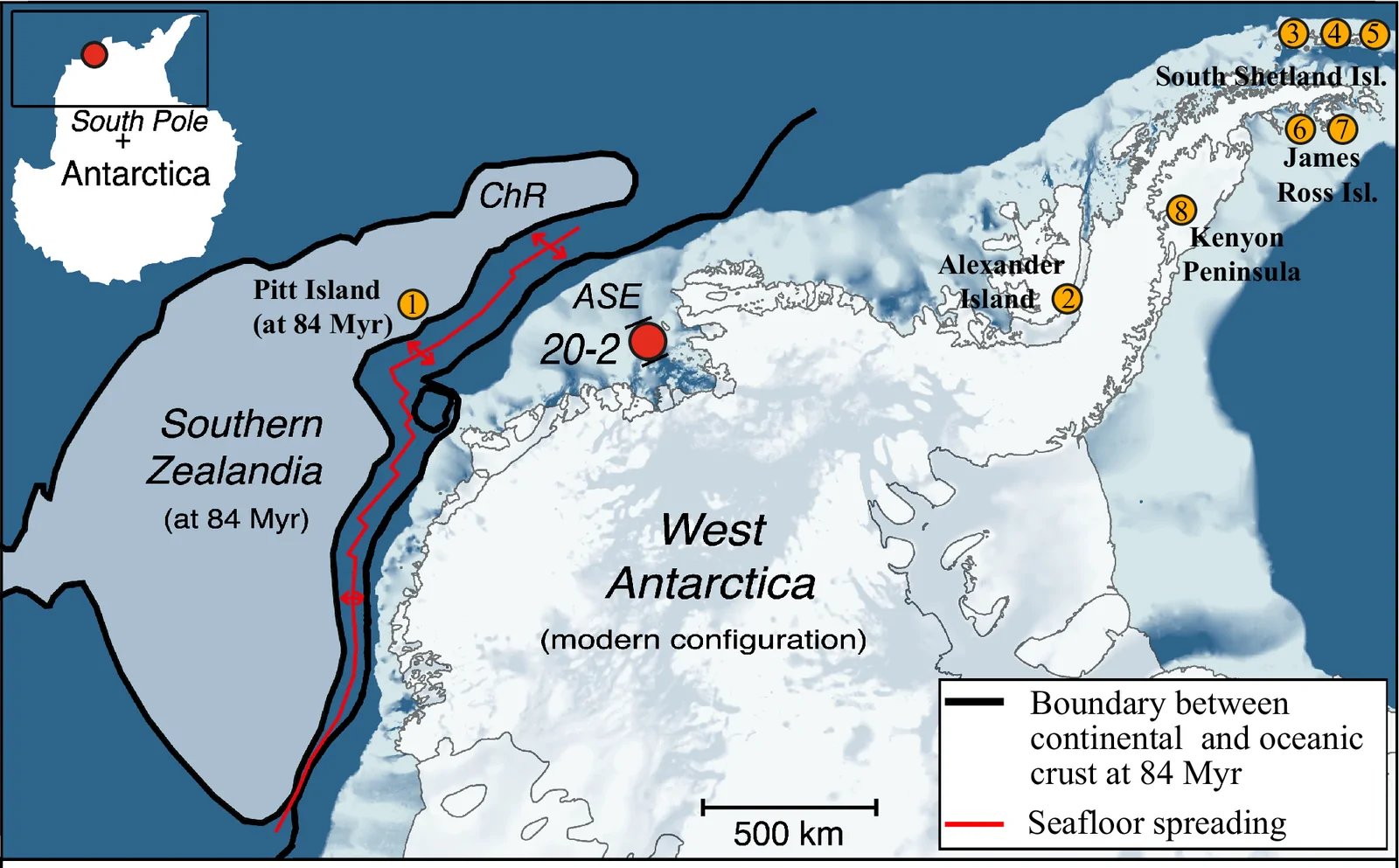
Location of drill site PS104 20-2 (red circle mark). The present-day location of West Antarctica is shown in relation to the reconstructed boundary between continental and oceanic crust at 84 Ma^35,36^. Orange circles mark the locations of other Turonian–Santonian (~93.9–83.6 Ma) sites with evidence for wildfires south of the polar circle, with numbers referring to: (1) Pitt Island^4^, (2) Alexander Island^5^, (3) Livingstone Island^6^, (4) King George Island^7^, (5) Nelson Island^8^, (6) and (7) James Ross Island^9,10^, (8) Kenyon Peninsula^11^. Modified from Klages et al.^12^.

Wildfires are a common global phenomenon, particularly during warm and carbon dioxide (CO_2_)-rich climates^1^. As one of the warmest and CO_2_-richest intervals of the past ~140 million years (Ma), the Late Cretaceous (100.5–66 Ma) is thought to have been prone to pronounced wildfires stoked by elevated atmospheric oxygen levels^2,3^ and high temperatures^1^. So far, however, Late Cretaceous wildfires were mostly reported from the Northern Hemisphere, whereas evidence for their extent and frequency in the Southern Hemisphere is still scarce, particularly beyond 60°S^2^. Frequent wildfires in Late Cretaceous (~99–90 Ma) temperate forests have been reported from the Chatham Islands, New Zealand^4^, which is yet the southernmost wildfire evidence about ~1500 km away from the South Pole (Fig. 1). Evidence for Late Cretaceous wildfires in Antarctica is very limited and confined to Alexander Island^5^, the South Shetland Islands^6–8^ and Antarctic Peninsula^9–11^. Volcanic activity has been proposed as the most likely ignition source for repeated large fires in the conifer-dominated forests of the Antarctic Peninsula^6–8^.

Here, we present a Late Cretaceous fire record from West Antarctica, documenting an increasing fire frequency within a developing *Sphagnum*-peat bog. Our study builds on a Turonian-Santonian (~92–83 Ma) root-bearing, fossiliferous mudstone sequence and lignite layer recovered from the West Antarctic continental shelf that revealed the presence of temperate, conifer-dominated rainforests only ~900 km from the South Pole^12^. We reconstruct early Antarctic fire ecology using macro- and microcharcoal, inertinite reflectance, and charcoal morphology along with lipid biomarkers and palynology. Our findings not only document the southernmost evidence of frequent, low-temperature wildfires, but also demonstrate their close association with the formation of the earliest known *Sphagnum*-peat bog from the southern high latitudes. Today, *Sphagnum* dominates high-latitude peatlands of the Northern Hemisphere forming an important sink of terrestrial carbon^13^, but their role as peat- and coal-forming elements has rarely been identified prior to the Quaternary^14^. Although *Sphagnum* fossils have been reported from the Middle Triassic onwards^15^, the peat moss remained a minor wetland component until the Cenozoic^16^. Phylogenetic reconstructions indicate that *Sphagnum* peat bogs diversified and expanded relatively late during the Miocene, supporting the assumption that Southern Hemisphere *Sphagnum* species might represent recent radiations from boreal-subarctic ancestors^17^.

## Results

### Regional setting, core stratigraphy, and vegetation change

**Fig. 2:**
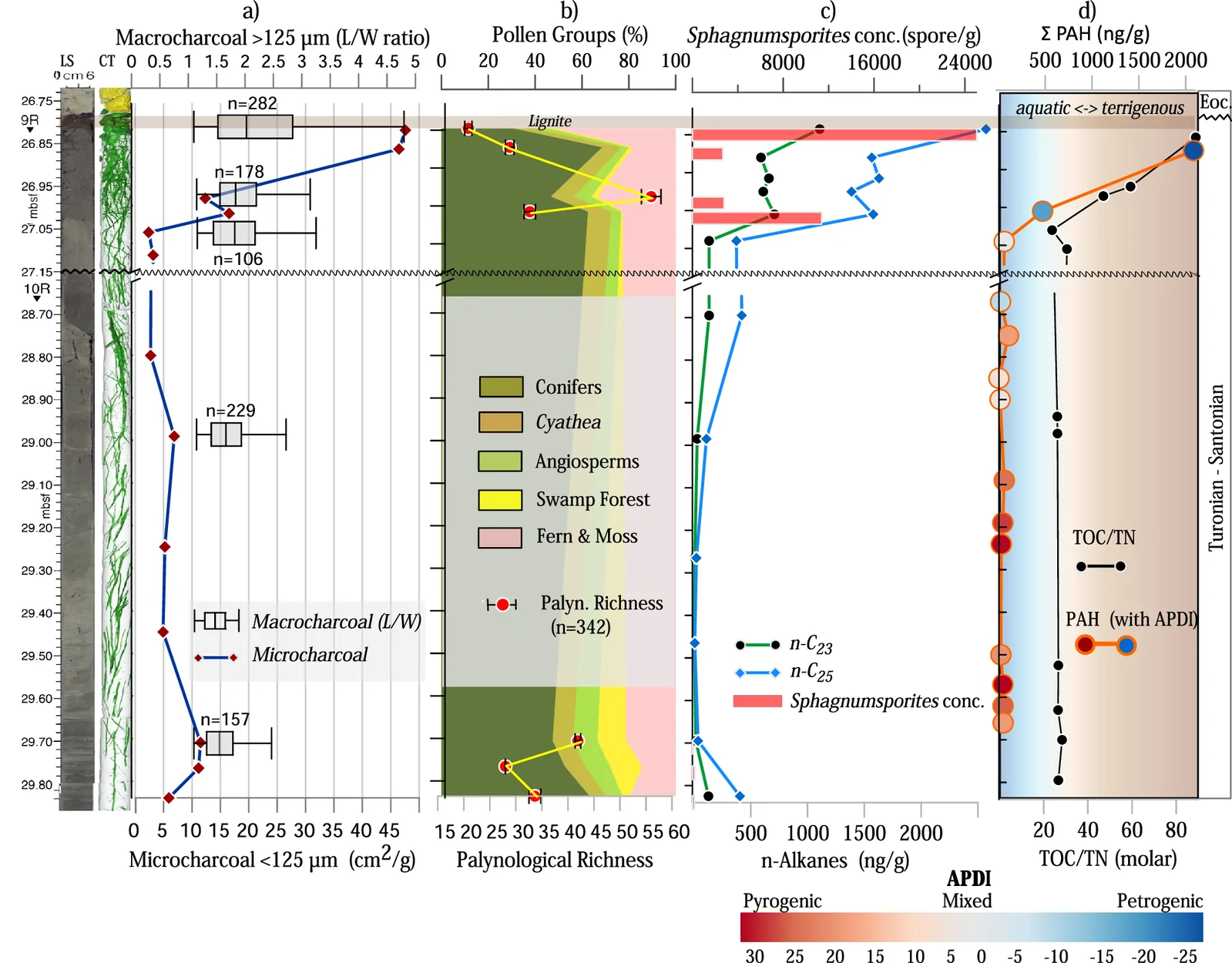
Palynology, biomarker and charcoal analyses. Linescan (LS) and X-ray computed tomography (CT) scan of the lowermost cores 9R and 10R of sediment record PS104_20-2, showing Late Cretaceous mudstone and lignite with roots (in green) and overlying Late Eocene sandstone (in yellow); **a** microcharcoal counts and length/width (L/W) ratio of macrocharcoal; **b** summative pollen percentage diagram and palynological richness estimated by rarefaction analysis (with 95% confidence intervals) as a proxy for plant biodiversity^57^; **c** *n*-alkanes and concentration of *Sphagnum*-type spores; **d** total organic carbon to total nitrogen (TOC/TN) ratios and concentration of polycyclic aromatic hydrocarbons (PAHs). PAH samples are coloured following the alkylated PAH derivative (APDI) index^31^. Red colours represent more pyrogenic inputs, blue colours represent more petrogenic inputs and white mixed inputs.

The sediment record was drilled at Site PS104_20 (73.57° S, 107.09° W; 946 m water depth) within the Pine Island cross-shelf trough in the Amundsen Sea Embayment (ASE), West Antarctica (Fig. 1), during RV *Polarstern* Expedition PS104^18^. The fossil- and charcoal-bearing sediments consist of a prominent, thin (5 cm) layer of indurated lignite fragments, separating the overlying Eocene sandstone unit^19,20^ from the underlying ≥3-m-thick laminated to stratified carbonaceous mudstone (Fig. 2). Palyno- and magnetostratigraphic dating indicates a Late Cretaceous (Turonian–Santonian; ~92–83 Ma) age for the mudstone sediment sequence^12^.

**Fig. 3:**
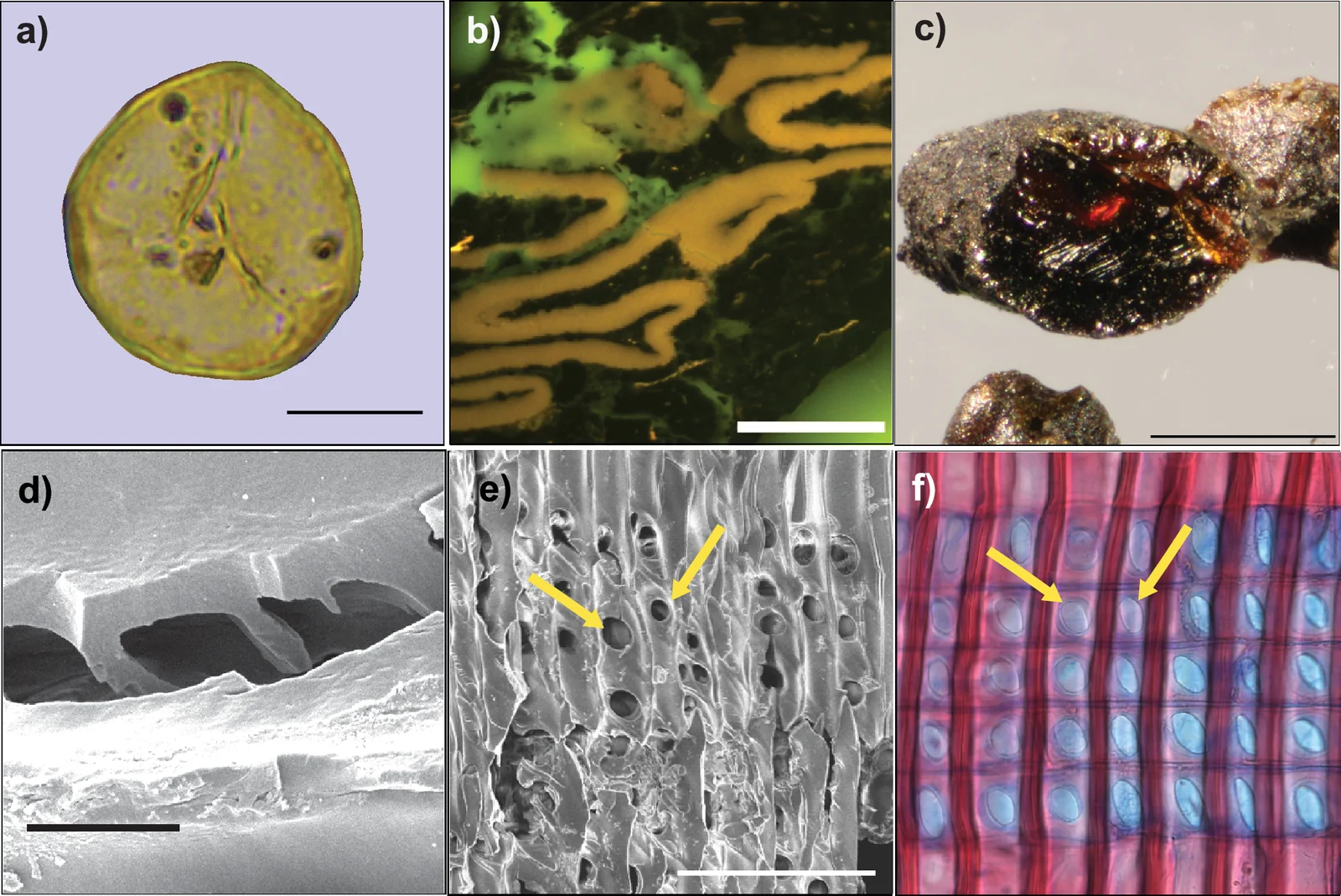
Photomicrographs of selected fossils within the lignite layer. **a** *Sphagnumsporites*spore; **b** megaspore maceral sporinite; **c** amber (Photo: Alexander Schmidt, University of Göttingen, Germany); **d** scanning electron micrograph (SEM) of ‘homogenised’ cell walls, indicative of charcoal burned at temperatures > 325 °C^26^ (see also Fig. 4); **e** SEM scan of radial wood section showing cross-field pits (gaps between longitudinal tracheids and wood ray cells), which have also been described as ‘window-like’ or ‘fenestriform’^29^ and are indicative of Podocarpaceae coniferous wood; **f** Radial wood section of modern *Lagarostrobos franklinii* showing morphological and anatomical similarities with fossil specimen (arrows indicate cross-field pits)^28^. Scale bar in (**a**, **d**) 10 µm; (**b**, **e**): 50 µm; (**c**) 500 µm.

The diverse palynological assemblage (Fig. 2 and Supplementary Tables) documents the predominance of austral temperate rainforests with Podocarpaceae and Araucariaceae conifer trees and abundant ferns, including the tree fern *Cyathea*, and few angiosperms (e.g. Proteaceae), which were undergoing major diversification and radiation during the Cretaceous. Abundant pollen percentages of *Nyssapollenites chathamicus* and *Taxodiaceaepollenites hiatus* in the lower part of the Late Cretaceous section, followed by a sharp increase in *Sphagnumsporites* (synonym *Stereisporites antiquasporitis*) and fern spores (e.g. *Laevigatosporites ovatus*) in the upper section, indicate a vegetation change from a riverine *Nyssapollenites*-*Taxodium* swamp forest towards a *Sphagnum*-peat bog. However, it should be noted that, despite its morphological similarity to *Nyssa* pollen, the nearest living relative of the Cretaceous taxon *Nyssapollenites chathamicus* remains unknown and likely unrelated to the Northern Hemisphere Tupelo swamp tree (*Nyssa* spp.*)*. The presence of sporinites in the lignite layer (Fig. 3) indicates anoxic, waterlogged preservation conditions (sporinite content total 5.3% by volume). The establishment of an open, fern-rich *Sphagnum* mire coincides with an increase in C_23_ and C_25_ *n-*alkanes, a change towards higher total organic carbon to total nitrogen (TOC/TN) ratios, and a rapid decline in species richness towards the top of the record (Fig. 2). The C_31_ hopane αβ 22S/(22S + 22 R) ratio, a proxy for the thermal maturity of sedimentary sequences^21^ ranges from 0.07 to 0.24 (mean 0.14 ± 0.06) and argues for an immature nature of the sedimentary organic matter with little diagenetic overprinting. The C_31_ hopane ββ/(αβ + ββ) index, which in peats has been shown to correlate with pH^22^, indicates an acidic environment for the uppermost samples (mean pH 4.31 ± 0.61), characteristic of ombrotrophic peat bogs. A change of the hydrological regime is also indicated by a reduction of the siliciclastic sediment input from the lower ≥3-m-thick, laminated to stratified carbonaceous mudstone to the prominent thin (5 cm) layer of indurated lignite fragments. Increasing abundances of mid-chain length C_23_ and C_25_ *n*-alkanes have been linked to higher input from submerged and floating-leaved plants^23^, or to peat expansion dominated by *Sphagnum*^24^. The TOC/TN ratios consistently indicate terrestrial environmental conditions (values > 20) and increase in parallel with *n*-alkane concentrations and *Sphagnum* spore abundance, which we interpret as evidence for expanding *Sphagnum*-dominated peat bogs. This interpretation is supported by the characteristically high TOC/TN ratios of modern peat bogs (often exceeding 40-50) compared to swamps and fens^25^.

### Micro- and macroscopic charcoal analyses

Microscopic (<125 µm) and macroscopic (>125 µm) charcoal particles are abundant throughout the entire record (Fig. 2a), and their number rapidly increases in the upper core 9R of sediment record PS104_20-2, suggesting a higher fire frequency and/or intensity^26^. This is corroborated by the frequent occurrence of the maceral fusinite (21.1% by volume) in the uppermost lignite layer. Many macrocharcoal particles exceed a length of 0.5–1 mm, indicating a local origin. The aspect ratio (length/width) of macrocharcoal particles slightly increases towards the top in core 9R (Fig. 2). The wider range might reflect a change in the fuel source with higher woodland contribution in the lower part^27^. However, higher aspect ratios might also be caused by the shift towards peatlands, which show an overall better preservation potential for elongated and fragile charcoal particles^27^. The fragments of carbonified conifer wood show some diagnostic features, such as bordered pits on tracheids and ‘window-like’ (fenestriform) cross-field pitting, that are characteristic of Podocarpaceae^28,29^ (Fig. 3).

### Polycyclic aromatic hydrocarbons and inertinite reflectance

Concentrations of polycyclic aromatic hydrocarbons (PAHs), which provide evidence of incomplete combustion of organic matter^30^, increase along with microcharcoal particles and *n*-alkanes, towards the top of the record indicating higher fire frequency and/or an increase in local fires closer to the drill site (Fig. 2). Most prominent are the 3-ring PAHs phenanthrene (Phe) and anthracene (Ant), together with the vascular plant biomarker retene (Ret), and the 4-ring PAHs fluoranthene (Fl) and pyrene (Py). Other abundant PAHs include perylene (Per), various benzopyrenes, including Bezo[a]pyrene (BaP) and Benzo[e]pyrene (BeP), as well as dimethylphenanthrenes (DMP). The alkylated PAH derivative index (APDI), which has been frequently used to distinguish between petrogenic and pyrogenic sample inputs^31,32^, indicates an origin from biomass burning for the majority of samples at mid to lower depths (Fig. 2d). A higher petrogenic influence at the top of core 9R reflect an increasing overprint from the uppermost lignite layer. Nevertheless, frequent biomass burning remains evident from abundant micro- and macrocharcoal and the maceral fusinite in the lignite layer (Figs. 2 and 3d).

**Fig. 4:**
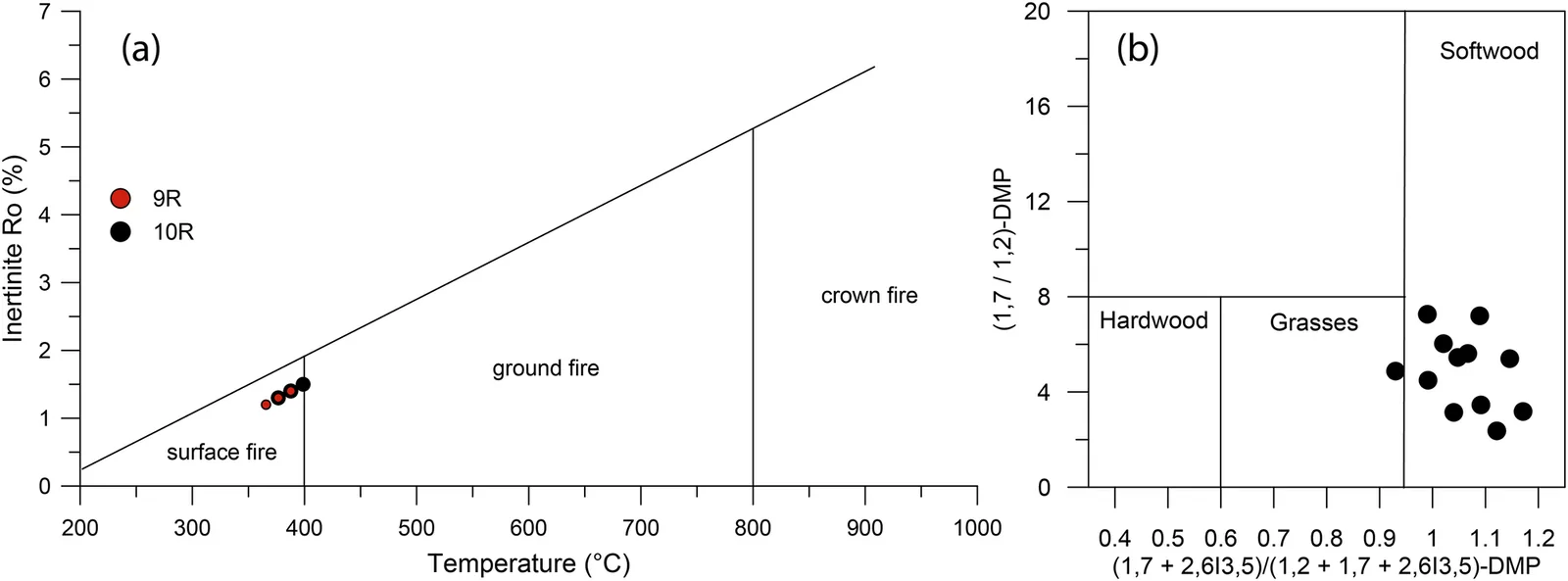
Fire-dependent parameters in our West Antarctic sediment record. **a** Reconstructed fire temperature based on inertinite reflectance values, indicating the presence of surface fires in the peatlands. **b** Distribution of dimethylphenanthrene isomers suggesting the burning of primarily softwood, such as gymnosperms.

The continuous presence of DMP in core 10R indicates a regular occurrence of wildfires, while a DMP-x ratio between 0.93 and 1.17 is indicative of softwood burning, suggesting conifers as the primary fuel source^31,33^ (Fig. 4). Inertinite reflectance (Ro %) values (<2) suggest that the charcoal primarily resulted from surface fires burning at low temperatures between ca. 370 and 400 °C^34^, whereby the uppermost layers of the record show slighly lower temperatures (Fig. 4). The combined increase in charcoal and PAHs therefore indicates a higher fire frequency rather than fire intensity.

## Discussion

### Wildfires near the South Pole

The presence of charcoal particles and PAHs throughout the entire record indicates that the temperate rainforests that grew near the South Pole under a warm Cretaceous greenhouse climate were prone to frequent forest fires. The opening of the West Antarctic rift system since the mid to Late Cretaceous resulted in major tectonic activity and magmatic events^35,36^. The proximity of the palaeosite to the active rift zone between Zealandia and West Antarctica points to volcanic eruptions as a potential ignition source for forest fire events (Fig. 1). Frequent forest fires ignited by volcanic eruptions during the Late Cretaceous have been previously proposed for the Antarctic Peninsula^6–8^. At the South Shetland Islands, fossil charcoalified woods of Podocarpaceae embedded in tuffite beds indicate intense and recurring wildfires, most likely ignited by pyroclastic flows^7,8^. Fossil evidence further suggests that prolonged exposure to hot volcanic ash drove fire temperatures as high as 900 °C^7^. Although magmatic activity along the West Antarctic Rift System was widespread, the nearest evidence of mid-Cretaceous volcanism to our drill site has been reported from the Jones Mountains, ~400 km from site PS104-20^37^. Whereas the persistent occurrence of charcoal and PAHs throughout our entire record demonstrates that fire was an intrinsic part of the Antarctic forest ecosystem, geochemical indicators of low-temperature surface burning are inconsistent with volcanism or pyroclastic flows as the primary ignition source.

In modern high-latitude conifer forests, lightning strikes represent the primary natural ignition source for wildfires^38^. For our temperate rainforest site, quantitative climate estimates from palynological data indicate an annual precipitation of ~1120 mm ^1,12^. We suggest that, despite of the overall humid climate, a seasonal fire regime was promoted by a combination of elevated atmospheric O₂ concentrations^1–3^, strong seasonal temperature changes, and potential monsoonal rainfall patterns during the Late Cretaceous. Seasonal temperature regimes, amplified by extreme seasonal variations in light availability, have previously been inferred for the Antarctic Cretaceous from palaeobotanical wood and fossil leaf analyses^39,40^. More recently, climate modelling studies demonstrated strong monsoonal conditions along the Antarctic margin during the warm, ice-free greenhouse climate of the early Eocene^41^. These simulations indicate that along the West Antarctic coast, precipitation was strongest in austral winter, likely creating dry, fire-prone conditions in its coastal swamps during summer.

**Fig. 5:**
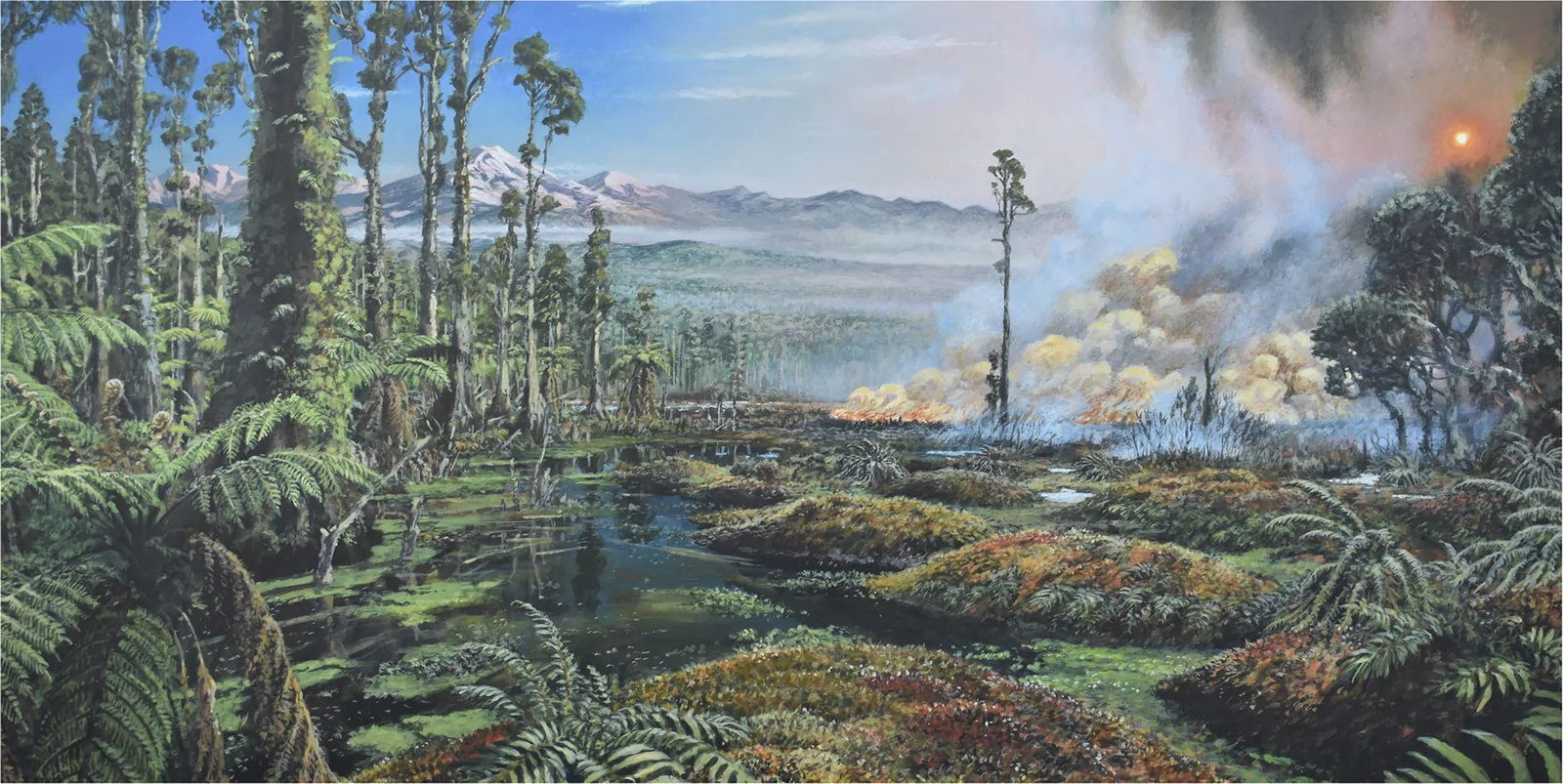
Artist’s impression of seasonal fires in fern-rich *Sphagnum*-peatlands and temperate rainforests in West Antarctica during the Turonian-Santonian. The painting is based on palaeofloral and environmental information inferred from palynological, geochemical, sedimentological and biomarker data obtained from cores 9R and 10R at site PS104_20. Original size of painting: 83.8 × 41.5 cm. J. McKay; this image is available under Creative Commons licence CC-BY 4.0.

The presence of micro- and macroscopic charcoal indicates both local fire input and long-distance windblown transport from regional sources^26^. The local fire regime was characterised by surface fires burning at low to moderate temperatures (Figs. 4 and 5), which are common in fossil and modern peats, wetlands and swampy forests^42–44^. These fires likely damaged tree bark, stimulating abundant resin production. Deposits of amber are frequently associated with fossil charcoal and notably also occur in the uppermost lignite layer of our record^45^ (Fig. 2). DMP ratios indicate that the charcoal originates predominantly from softwood combustion, consistent with palynological evidence for conifer-dominated forests. SEM analyses of macrocharcoal particles from the uppermost lignite layer indicate that the larger charcoal particles originate from Podocarpaceae conifers, which also provide 34–61% of the total pollen and spore influx and were a dominant part of the regional temperate forests in Antarctica during the Late Cretaceous. Modern species of the podocarp family which show „window-like” (fenestriform) cross-field pitting and grow in waterlogged, peaty habitats include *Lagarostrobos franklinii* (Huon pine) and *Manoao colensoi* (silver pine). While *Manoao* pollen cannot be distinguished morphologically from other species of Podocarpaceae, the occurrence of the pollen taxon *Phyllocladidites mawsonii* likely indicates the presence of *Lagorostrobos* in the local vegetation (see Supplementary Tables).

### *Sphagnum*-peat and changing fire regimes

Local vegetation along the Pacific margin of West Antarctica transitioned from a *Nyssapollenites*-*Taxodium* swamp forest to a *Sphagnum*-rich peat bog, mirroring modern hydroseral successions that progress from open water and swamp to a peat bog. With percentages exceeding 36% of the total sum of pollen and spores (or >25,000 spores per gram of sediment) (Fig. 2), our Antarctic sediment record provides the earliest evidence of a coal-forming *Sphagnum*-peat bog. Although the peak abundance of *Sphagnum* spores coincides with the highest concentrations of C_23_ and C_25_ *n*-alkanes, we observe a decline in the C_23_/C_31_ and C_23_/C_29_ *n*-alkane ratios (Supplementary Tables), which have previously been proposed as indicators distinguishing *Sphagnum*-dominated peat from grass- and wood-dominated material^46,47^. In our record, these ratios do not appear to reliably reflect the pronounced *Sphagnum* maximum indicated by the spore assemblage. We therefore infer that the expansion of higher land plants, contributing predominantly long-chain *n*-alkanes, likely overprinted the *Sphagnum*-specific C_23_ and C_25_ signals. Nevertheless, the marked peak in *Sphagnum* spores clearly demonstrates its dominance during this interval.

In the floristically similar Late Cretaceous Tupuangi Formation, New Zealand, *Sphagnum*-type spores (*Stereisporites*) occur only sporadically (<1%)^48^. One of the earliest known coal-forming *Sphagnum* peats dates to the early Eocene (~55 Ma) ‘Schöningen lignite’, NW Germany, almost 35 Myr younger than our West Antarctic record^14,46,49^. Despite marked floristic differences, the Eocene lignite shows some striking similarities to our Antarctic record in documenting vegetation successions from a *Nyssa*-*Taxodium* riverine swamp to a *Sphagnum* dominated peat bog. Both peatlands were rich in ferns, as suggested by abundant *Laevigatosporites* spores (Supplementary Tables). As in our record, increased *Sphagnum* abundance correlates with elevated charcoal, indicating regular wildfire activity within peat bogs, and declining biodiversity (Fig. 2), potentially reflecting increasingly oligotrophic and nutrient-poor conditions in rain-fed, ombrotrophic mires^42,49^. This change coincides with a lower pH indicating acidic environmental conditions that are typical for ombrotrophic peat bogs^22^. The spores also show close morphological similarities to *Sphagnumsporites*-type spores identified alongside *Sphagnum* leaves in the early Eocene lignite deposits of NW Germany^49^. Our reconstruction of a Cretaceous *Sphagnum* peat bog on Antarctica therefore challenges the hypothesis that Southern Hemisphere taxa derive from a late radiation from boreal-subarctic ancestors^17^.

Close links between cyclic forest to peatland successions and associated fire activity have also been reported from the Oligocene to Miocene brown coals of the Latrobe Valley, Australia^43^. For both Schöningen and Latrobe Valley, local vegetation changes have been interpreted as hydroseral successions (terrestrialisation) within wetland settings rather than regional climate shifts^43,50^.

Age constraints for our West Antarctic sediment record preclude determination of the temporal span of recorded environmental changes. However, the composition of the regional Podocarpaceae and Araucariaceae coniferous forests remains largely unchanged suggesting climatic stability throughout the recorded interval. In contrast, variations in *Nyssapollenites*, Cupressaceae, ferns and mosses reflect local changes in vegetation and fire ecology, driven by peat aggradation and changes in water and nutrient availability rather than climate. The near-coastal location of our drill site (Fig. 1) suggests that sea-level fluctuations and subsequent changes in the coastline may have triggered this succession by changing the hydrological regime.

The early Late Cretaceous record demonstrates that recurrent fire constituted an intrinsic element of *Sphagnum* peatland ecology in West Antarctica. Fire disturbance probably functioned as a natural ecosystem driver by hampering tree growth and promoting fern establishment, hence maintaining an open vegetation structure. These findings align with more recent palaeoecological studies from the northern high latitudes, indicating that, over the last 10,000 years, fire operated as a fundamental disturbance regime across diverse wetland and peatland systems^50,51^.

## Conclusions

Our sediment record from the West Antarctic mainland documents the southernmost evidence of frequent, low-temperature wildfires and demonstrates their intimate association with the formation of the earliest known *Sphagnum*-peat bog from southern high latitudes. This predates previous evidence of coal-forming *Sphagnum* peatlands by ~35 million years and questions the assumption of a recent radiation of *Sphagnum* from boreal-subarctic ancestors into the Southern Hemisphere^17^. As such, the record offers an exceptional opportunity to study the co-evolution of fire and peatland ecology in an ice-sheet-free greenhouse world with high atmospheric CO₂-concentrations.

Despite lacking modern floristic analogues and having developed under substantially higher atmospheric oxygen and CO₂-concentrations than present, our 90-million-year-old West Antarctic peatland exhibits ecological functions remarkably similar to those of modern systems. These include hydroseral succession, the dominant process that formed most ombrotrophic *Sphagnum* peat bogs in the Northern Hemisphere since the Last Glacial Maximum. Multiple proxies used in our study capture the full suite of processes characteristic of ombrotrophic *Sphagnum*-peat development, such as hydrological and edaphic change, and a local decline in plant biodiversity. As important global carbon sinks, contemporary peatlands face increasing fire vulnerability under projected climate change scenarios^52^, making insights from past warm greenhouse environments essential for predicting ecosystem trajectories and informing evidence-based conservation strategies.

## Methods

### Sample preparation

This study focuses on a drill record that recovered a ~3 m-long palynomorph-rich and root-bearing carbonaceous mudstone of early Late Cretaceous age in cores 9R and 10R^12^. The record was retrieved during RV *Polarstern* Expedition PS104 using the MARUM-MeBo70 seafloor drill rig as the bottom section of the ~30 m-long PS104_20-2 drill core from the mid-shelf section of Pine Island Trough in the eastern ASE, West Antarctica. Samples for microfossil and geochemical analyses were collected from multiple depths throughout cores 9R and 10R.

### Maceral composition and inertinite reflectance

Mean random inertinite reflectance measurements were performed on sediments recovered from cores 9R and 10R. All samples were molded and surface-impregnated with epoxy resin (Araldite 1020), after which they were freshly polished with the final polishing step being carried out using a 0.05 µm silica suspension. Reflectance measurements were conducted using a reflected-light microscope (Axio Imager M2m, Zeiss) equipped with a tungsten–halogen lamp (12 V, 100 W) for incident white light and an Epiplan-Neofluar 50×/1.0 oil-immersion objective. Data acquisition was performed using a BASLER CCD camera system coupled with DISKUS Fossil software version 5.0.7563 (Technisches Büro C. Hilgers). Calibration was carried out using an yttrium–aluminum–garnet (YAG) reflectance standard with a certified reflectivity of 0.889%. Mean random inertinite reflectance was determined at a wavelength of 546 nm using the 50× oil-immersion objective by measuring the proportion of incident light reflected from the surfaces of individual inertinite particles. >100 reflectance measurements were obtained from inertinite particles for each sample to ensure statistically robust mean values. Inertinite reflectance values were subsequently converted into combustion temperatures using the transfer function of Gao et al.^34^. Maceral analysis was conducted on polished particulate blocks according to ISO 7404-3:2009 using a Leica DM 4000 P microscope in incident light and fluorescence mode at a total magnification of 500x. Five hundred points were counted in total using an oil immersion objective of 50x and a semi-automatic point counter from PELCON Materials & Testing ApS, Søborg / Denmark. ICCP system 1994 lignite nomenclature was applied for maceral analyses^53–55^. The reflectance was measured on ulminite macerals according to ISO 7404-5:2009 by connecting the Leica DM 4000 P microscope to a UV-NIR photospectrometer from A.S. & Co. GmbH, Munich.

### Qualitative and quantitative charcoal and pollen analyses

Between 2 and 3 g of sediment from five samples across both cores PS104_9R and 10R were taken for micro- and macrocharcoal analyses. To avoid potential fragmentation of charcoal particles during preparation, the entire sediment sample was dissolved without crushing using standard palynological acid maceration techniques, including 30% HCl and 40% HF. *Lycopodium* marker spore tablets were added to each sample for quantitative analysis of microcharchoal particles using the point count method^56^. The samples were separated into three size classes by carefully sieving through a 125 µm and 10 µm mesh. The microcharcoal size fraction (10–125 µm) was mounted with glycerine gelly following final treatment with hot H_2_O_2_ (30%) for three hours to facilitate the separation of charcoal from other organic matter. The five microcharcoal samples and additional nine samples that have been previously prepared using the same acid maceration technique^12^ were counted using a Leica DM2000 microscope at 400x magnification. Rarefied palynological richness, as a proxy for changes in plant alpha-diversity through time^57^, was quantified using PAST 5.1^58^. Whereas the microcharcoal counts reveal information on past fire frequency and intensity, the aspect ratio (length/width) of macrocharcoal particles (>125 µm) provide information on the potential fuel source^27^. We analysed macrocharcoal particles using a Leica Stereomicroscope AFXX at ×50 magnification and determined the aspect ratio (length/width) semi-automatically using the open-source software ImageJ and the protocol by Ruiz-Perez et al.^59^. Before calculating the aspect ratio, all software-generated outline images of charcoal particles were manually checked for wrong identifications.

Charcoal samples from the lignite layer of core 9R-1W (26.8 m) and from the mudstone of core 10R-1W (28.99 m) were selected for wood identification. The wood anatomical structures of the charcoal specimens were microscopically investigated using a Keyence digital microscope VHX 7000 and Thermo Fisher Scientific Desktop Scanning Electron Microscope Phenom Pharos G 2. Wood structures were identified from literature^28,29^.

### Bulk geochemical sediment composition

Total carbon (TC) and total nitrogen (TN) contents were analysed with an Elementar Vario EL III elemental analyser. The TOC content was determined after removal of the total inorganic carbon (TIC) with 20% HCl using an ELTRA CS-2000. The carbonate (CaCO_3_) content was calculated by subtracting the TOC from the TC content and multiplying the difference (TIC) by 8.33; that is, the ratio between the molecular weights of CaCO_3_ and C. The TOC/TN ratio was calculated on a molar basis.

### Organic geochemistry

Freeze-dried and homogenised sediment samples were extracted by means of ultrasonication using a solvent mixture of dichloromethane (DCM):methanol (MeOH) (2:1, v/v). After centrifugation, the total lipid extract (TLE) was dried by rotary evaporation. The extraction was repeated twice. The combined TLEs were fractionated using silica open-column chromatography and *n*-hexane as eluent to obtain aliphatic lipids. *n*-Alkanes were analysed using an HP 6890 gas chromatograph equipped with a DB-5MS capillary column (30 m × 0.25 mm inner diameter, 0.25 μm film thickness) and a flame ionisation detector with the oven temperature programmed as follows: 60 °C (held for 3 min), increase to 150 °C (20 °C/min) and then to 320 °C (6 °C/min; held for 40 min). The identification of *n*-alkanes was based on comparison of their retention times with those of reference compounds that were run on the same instrument. Aliquots of the *n*-hexane fraction were also analysed for the presence and distribution of hopanes using a Mega series HRGC 5160 gas chromatograph (Carlo Erba, Italy) fitted with a Phenomenex ZB-5 column (30 m × 0.25 mm inner diameter, 0.25 μm film thickness) coupled to a Trace MS (Thermoquest). Helium was used as carrier gas with a constant flow of 1 ml/min. The oven temperature was raised from 80 °C to 160 °C at 10 °C/min, followed by 3 °C/min to a final temperature of 320 °C. The mass spectrometer was operated in electron impact ionisation (EI+) mode with an electron energy of 70 eV and an ion source temperature of 200 °C. Hopanes were identified by integrated comparison using *m/z* 191. We applied the C_31_ hopane αβ 22S/(22S + 22 R) ratio as a proxy to determine the thermal maturity of the organic matter^21^ and the C_31_ hopane ββ/(αβ + ββ) index as a peat-specific proxy to reconstruct fossil pH conditions^22^.

### Polycyclic aromatic hydrocarbon analysis

Fifteen sediment samples, including a fragment of the lignite layer, were analysed for the presence of polycyclic aromatic hydrocarbons (PAHs), indicative of the incomplete combustion of organic matter and frequently used to trace the occurrence of wildfires in the geological record^32^. Freeze-dried and homogenised sediments were extracted repeatedly (at least six times) in an ultrasonic bath with a solvent mixture of DCM:MeOH (9:1, v:v). The TLEs were separated into aliphatic, aromatic and polar fractions using *n*-hexane, DCM:*n*-hexane (2:1, v:v) and MeOH as eluents, respectively. The aromatic fractions, containing PAHs, were analysed using a Thermo Scientific Trace 1310 gas chromatograph coupled to a Thermo Fisher TSQ 8000 Evo single quadrupole mass spectrometer. Samples were injected on-column on a Phenomenex DB-5MS (25 m × 0.25 mm i.d., 0.25 µm film thickness). Helium was used as carrier gas. The oven programme was as follows: 60 °C (held 3 min), ramped to 310 °C at 3 °C/min and then held isothermal for 20 min. The mass spectrometer was operated at an electron energy of 70 eV and scanning *m/z* 50-800 with a scan time of 1.0 s. Identification of PAHs was based on authentic standards, published mass spectra and retention times. Quantification of PAHs was achieved by adding a deuterated d_34_-hexadecane standard at a concentration of 6.03 ng/µl to each sample. The APDI was calculated as reported previously^21^ and used to distinguish between petrogenic and pyrogenic source inputs.

## Supplementary information

Transparent Peer Review file

Description of Additional Supplementary table file

Supplementary Tables

## Data Availability

All data analysed in this study are available in Supplementary Tables. All relevant data are available from the corresponding authors upon request and online^60^ via PANGAEA at https://doi.org/10.1594/PANGAEA.994798.

## References

[1] Bowman, D. M. J. S. et al. Fire in the Earth system. *Science* **324**, 481–484 (2009). 19390038 10.1126/science.1163886

[2] Brown, S. A. E., Scott, A. C., Glasspool, I. J. & Collinson, M. E. Cretaceous wildfires and their impact on the Earth system. *Cretac. Res.* **36**, 162–190 (2012).

[3] Lü, D.-W. et al. A synthesis of the Cretaceous wildfire record related to atmospheric oxygen levels? *J. Palaeogeogr.* **13**, 149–164 (2024).

[4] Mays, C., Cantrill, D. & Bevitt, J. Polar wildfires and conifer serotiny during the Cretaceous global hothouse. *Geology* **45**, 1119–1122 (2017).

[5] Falcon-Lang, H. J., Cantrill, D. J. & Nichols, G. J. Biodiversity and terrestrial ecology of a mid-Cretaceous, high-latitude floodplain, Alexander Island, Antarctica. *J. Geol. Soc.* **158**, 709–724 (2001).

[6] Kostova, I. et al. Characterization of organic matter from the Cretaceous sedimentary and volcano-sedimentary strata from Livingston Island, Antarctic Peninsula: insights from organic petrology, molecular proxies and carbon and hydrogen isotopes. *Int. J. Coal Geol.* **252**, 103940 (2022).

[7] Manfroi, J. et al. “Antarctic on fire”: paleo-wildfire events associated with volcanic deposits in the Antarctic Peninsula during the Late Cretaceous. *Front. Earth Sci.* **11**, 1–11 (2023).

[8] Manfroi, J., Dutra, T. L., Gnaedinger, S., Uhl, D. & Jasper, A. The first report of a Campanian palaeo-wildfire in the West Antarctic Peninsula. *Palaeogeogr. Palaeoclimatol. Palaeoecol.* **418**, 12–18 (2015).

[9] Lima, F. J. d. et al. Wildfires in the Campanian of James Ross Island: a new macro-charcoal record for the Antarctic Peninsula. *Polar Res.* **40**, https://api.semanticscholar.org/CorpusID:239463377 (2021).

[10] Kvaček, J. & Sakala, J. Late Cretaceous flora of James Ross Island (Antarctica) – preliminary report. *Czech Polar Rep.* **1**, 96–103 (2012).

[11] Eklund, H., Cantrill, D. J. & Francis, J. E. Late Cretaceous plant mesofossils from Table Nunatak, Antarctica. *Cretac. Res.* **25**, 211–228 (2004).

[12] Klages, J. P. et al. Temperate rainforests near the South Pole during peak Cretaceous warmth. *Nature* **580**, 81–86 (2020). 32238944 10.1038/s41586-020-2148-5

[13] Gorham, E. Northern peatlands: role in the carbon cycle and probable responses to climatic warming. *Ecol. Appl.* **1**, 182–195 (1991). 27755660 10.2307/1941811

[14] Riegel, W. & Wilde, V. An early Eocene *Sphagnum* bog at Schöningen, northern Germany. *Int. J. Coal Geol.* **159**, 57–70 (2016).

[15] Pant, D. D. & Basu, N. On two structurally preserved bryophytes from the Triassic of Nidpur, India. *J. Palaeosci.* **25**, 340–352 (1976).

[16] Greb, S. F., DiMichele, W. A. & Gastaldo, R. A. *Evolution and importance of wetlands in earth history, in Wetlands through Time* (eds. S.F. Greb & W.A. DiMichele) 0 (Geological Society of America, 2006).

[17] Shaw, A. J. et al. Peatmoss (*Sphagnum*) diversification associated with Miocene Northern Hemisphere climatic cooling? *Mol. Phylogenet. Evol.* **55**, 1139–1145 (2010). 20102745 10.1016/j.ympev.2010.01.020

[18] Gohl, K. et al. MeBo70 Seabed Drilling on a Polar Continental Shelf: Operational Report and Lessons From Drilling in the Amundsen Sea Embayment of West Antarctica. *Geochem. Geophys. Geosyst.* **18**, 4235–4250 (2017).

[19] Zundel, M. et al. A large-scale transcontinental river system crossed West Antarctica during the Eocene. *Sci. Adv.* **10**, eadn6056 (2024). 38838149 10.1126/sciadv.adn6056 PMC11152126

[20] Marschalek, J. W. et al. Reconstructing Eocene Antarctic river drainage from provenance analysis of Amundsen Sea embayment sediments. *Sci. Adv.* **11**, eaea2373 (2025). 41370375 10.1126/sciadv.aea2373 PMC12693975

[21] Seifert, W. K. & Moldowan, J. M. The effect of thermal stress on source-rock quality as measured by hopane stereochemistry. *Phys. Chem. Earth* **12**, 229–237 (1980).

[22] Inglis, G. N. et al. Distributions of geohopanoids in peat: implications for the use of hopanoid-based proxies in natural archives. *Geochim. Cosmochim. Acta* **224**, 249–261 (2018).

[23] Ficken, K. J., Li, B., Swain, D. L. & Eglinton, G. An n-alkane proxy for the sedimentary input of submerged/floating freshwater aquatic macrophytes. *Org. Geochem.* **31**, 745–749 (2000).

[24] Baas, M., Pancost, R., van Geel, B. & Sinninghe Damsté, J. S. A comparative study of lipids in *Sphagnum* species. *Org. Geochem.* **31**, 535–541 (2000).

[25] Watmough, S. et al. Variation in carbon and nitrogen concentrations among peatland categories at the global scale. *PLOS ONE* **17**, e0275149 (2022). 36417456 10.1371/journal.pone.0275149 PMC9683585

[26] Scott, A. C. Charcoal recognition, taphonomy and uses in palaeoenvironmental analysis. *Palaeogeogr. Palaeoclimatol. Palaeoecol.* **291**, 11–39 (2010).

[27] Crawford, A. J. & Belcher, C. M. A Morphological Analysis of Holocene Charcoal Particles From a Peatland in Southwest England. *Mires Peat* **28**, 33 (2022).

[28] Heinz, I. Systematische Erfassung und Dokumentation der mikroanatomischen Merkmale der Nadelhölzer aus der Klasse der Pinatae. Ph.D. Thesis, Technische Universität München, Munich, Germany, 2004; p. 209.

[29] IAWA Committee. List of microscopic features for softwood identification. *IAWA J.* **25**, 1–70 (2004).

[30] Yunker, M. B. et al. PAHs in the Fraser River basin: a critical appraisal of PAH ratios as indicators of PAH source and composition. *Org. Geochem.* **33**, 489–515 (2002).

[31] Karp, A. T., Holman, A. I., Hopper, P., Grice, K. & Freeman, K. H. Fire distinguishers: refined interpretations of polycyclic aromatic hydrocarbons for paleo-applications. *Geochim. Cosmochim. Acta* **289**, 93–113 (2020).

[32] Lyons, S. L. et al. Organic matter from the Chicxulub crater exacerbated the K–Pg impact winter. *Proc. Natl. Acad. Sci. USA* **117**, 25327–25334 (2020). 32989138 10.1073/pnas.2004596117 PMC7568312

[33] Kappenberg, A., Braun, M., Amelung, W. & Lehndorff, E. Fire condensates and charcoals: Chemical composition and fuel source identification. *Org. Geochem.* **130**, 43–50 (2019).

[34] Gao, D. et al. Inertinite reflectance in relation to combustion temperature. *Processes* **12**, 2452 (2024).

[35] Wobbe, F., Gohl, K., Chambord, A. & Sutherland, R. Structure and breakup history of the rifted margin of West Antarctica in relation to Cretaceous separation from Zealandia and Bellingshausen plate motion. *Geochem. Geophys. Geosyst.* **13**, Q04W12 (2012).

[36] Jordan, T. A., Riley, T. R. & Siddoway, C. S. The geological history and evolution of West Antarctica. *Nat. Rev. Earth Environ.* **1**, 117–133 (2020).

[37] Pankhurst, R. J., Millar, I. L. & Grunow, A. M. Sotrey B.C. The Pre-Cenozoic magmatic history of the Thurston Island Crustal Block, west Antarctica. *J. Geophys. Res. Solid Earth***ume 98**, 11835–11849 (1993).

[38] Engle, B., Bratoev, I., Crowley, M. A., Zhu, Y. & Senf, C. Distribution and characteristics of lightning-ignited wildfires in boreal forests – the BoLtFire database. *Earth Syst. Sci. Data* **17**, 2249–2276 (2025).

[39] Francis, J. E. & Poole, I. Cretaceous and early Tertiary climates of Antarctica: evidence from fossil wood. *Palaeogeogr. Palaeoclimatol. Palaeoecol.* **182**, 47–64 (2002).

[40] Jacques, F., Shi, G., Li, H. & Wang, W.-M. An early–middle Eocene Antarctic summer monsoon: Evidence of ‘fossil climates. *Gondwana Res.* **25**, 1422–1428 (2014).

[41] Baatsen, M., Bijl, P., von der Heydt, A., Sluijs, A. & Dijkstra, H. Resilient Antarctic monsoonal climate prevented ice growth during the Eocene. *Climate* **20**, 77–90 (2024).

[42] Robson, B. E. et al. Early Paleogene wildfires in peat-forming environments at Schöningen, Germany. *Palaeogeogr. Palaeoclimatol. Palaeoecol.* **437**, 53–62 (2015).

[43] Korasidis, V. A. et al. Cyclic floral succession and fire in a Cenozoic wetland/peatland system. *Palaeogeogr. Palaeoclimatol. Palaeoecol.* **461**, 237–252 (2016).

[44] Malone, S. L., McLeod, G., Chen, A. & Tupaj, M. Contemporary fire regimes of the subtropical Everglades. *Sci. Data* **12**, 1622 (2025). 41057383 10.1038/s41597-025-05917-6 PMC12504416

[45] Klages, J. P. et al. First discovery of Antarctic amber. *Antarct. Sci.* **36**, 439–440 (2024).

[46] Inglis, G. N. et al. Ecological and biogeochemical change in an early Paleogene peat-forming environment: linking biomarkers and palynology. *Palaeogeogr. Palaeoclimatol. Palaeoecol.* **438**, 245–255 (2015).

[47] Bingham, E. M. et al. Conservative composition of n-alkane biomarkers in Sphagnum species: implications for palaeoclimate reconstruction in ombrotrophic peat bogs. *Org. Geochem.* **41**, 214–220 (2010).

[48] Mays, C. A Late Cretaceous (Cenomanian–Turonian) south polar palynoflora from the Chatham Islands, New Zealand. *Mem. Assoc. Australas. Palaeontol.* **47**, 1–92 (2015).

[49] Lenz, O. K., Riegel, W. & Wilde, V. Greenhouse conditions in lower Eocene coastal wetlands? -Lessons from Schöningen, Northern Germany. *PLOS ONE* **16**, e0232861 (2021). 33439859 10.1371/journal.pone.0232861 PMC7806146

[50] Feurdean, A. et al. Holocene wildfire regimes in western Siberia: interaction between peatland moisture conditions and the composition of plant functional types. *Climate* **18**, 1255–1274 (2022).

[51] Sim, T. G. et al. Regional variability in peatland burning at mid-to high-latitudes during the Holocene. *Quat. Sci. Rev.* **305**, 108020 (2023).

[52] Turetsky, M. R. et al. Global vulnerability of peatlands to fire and carbon loss. *Nat. Geosci.* **8**, 11–14 (2015).

[53] ICCP The new inertinite classification (ICCP System 1994). *Fuel* **80**, 459–471 (2001).

[54] Pickel, W. et al. Classification of liptinite – ICCP System 1994. *Int. J. Coal Geol.* **169**, 40–61 (2017).

[55] Sýkorová, I. et al. Classification of huminite—ICCP System 1994. *Int. J. Coal Geol.* **62**, 85–106 (2005).

[56] Clark, R. L. Point count estimation of charcoal in pollen preparation and thin sections of sediments. *Pollen et. Spores* **24**, 523–535 (1982).

[57] Birks, H. J. B. & Line, J. M. The use of rarefaction analysis for estimating palynological richness from quaternary pollen-analytical data. * Holocene* **2**, 1–10 (1992).

[58] Hammer, O., Harper, D. & Ryan, P. PAST: paleontological statistics software package for education and data analysis. *Palaeontol. Electron.* **4**, 1–9 (2001).

[59] Ruiz-Pérez, J., Aleman, J. C. & Veldman, J. W. Reproducible protocol for the extraction and semi-automated quantification of macroscopic charcoal from soil. *PLoS ONE* **19**, e0304198 (2024). 38995962 10.1371/journal.pone.0304198 PMC11244820

[60] Salzmann, U. et al. Charcoal, palynology and geochemical analyses of core 9R and 10R from MARUM-MeBo70 Site PS104_20-2 [dataset bundled publication]. PANGAEA, 10.1594/PANGAEA.994798 (2026).

