# Supplementary material for: Co-evolution of wildfires and early Sphagnum-peatlands near the Cretaceous South Pole
Source: Commun Earth Environ. 2026 Sep 7;7(1):715. doi: 10.1038/s43247-026-03959-1 (PMC13549950; doi:10.1038/s43247-026-03959-1)
Supplement: Supplementary file 1 [file 43247_2026_3959_MOESM1_ESM.pdf]

nature portfolio

Peer Review File

Co-evolution of wildfires and early Sphagnum-peatlands near the Cretaceous South Pole

Corresponding Author: Professor Ulrich Salzmann

This file contains all reviewer reports in order by version, followed by all author rebuttals in order by version.

Version 0:

Reviewer comments:

Reviewer #1

(Remarks to the Author)
This is a well-written manuscript that provides the oldest known occurrence of a "modern-type" Sphagnum-dominated wetland in the geological record (as far as I am aware!). This pushes the oldest known occurrence back by 35 million years and thus will be of interest to the (paleo)ecological and (paleo)botanical community. The team use a wide variety of analysis to reach their conclusions - I do have a few comments about the biomarker interpretation but this does not affect the overall interpretation.

I have a couple of areas that I would like the authors to explore in more detail - but otherwise, I think this is a strong manuscript that would be well suited to this journal and be of interest to the Mesozoic and Cenozoic climate community.

1) Broader introduction

The introduction was concise and well written – but there are two areas that the authors may wish to discuss further.

First, it is worth noting the oldest occurrence of a "modern" Sphagnum-type bog in the geological record. By explaining that these records are scarce and rarely found beyond the Eocene (~55 Ma), it makes your conclusions more impactful. Second, perhaps the evolutionary aspects need to be discussed - when did Sphagnum evolve in the geological record? This paper (https://www.sciencedirect.com/science/article/pii/S1055790310000229#fig1) provides a nice overview of radiation of southern and northern hemisphere taxa

2) n-alkane data

It is interesting that there is an increase in C23 and C25 n-alkanes (putative sphagnum biomarkers) alongside an increase in sphagnum pollen – but what about other long-chain n-alkanes? Do they also increase? If they all increase, it might just suggest more plant input, rather than a shift in the type of plants.

The C23/C31 and C23/C29 n-alkane ratio has been proposed as a tracer for Sphagnum input into ancient peat deposits (see Nott 2000, Bingham 2010 and Bush and McInerey 2013). A number of studies have shown the close correspondence between these ratios and the relative abundance of Sphagnum leaves in a variety of modern bogs, but rarely applied in past – this ratio should therefore be added and the authors should evaluate whether this remains a useful chemotaxonomic tool in Cretaceous samples. When we applied this approach in the Schoningen early Eocene lignite, you see a strong increase in C23/C31 ratio from ~1 to ~7 alongside an increase in the sphagnum spores.

If your ratio does not change, that is fine - you clearly have loads of sphagnum pollen and I would not doubt the overall conclusions - but its useful for biomarker community to know how trustworthy this ratio is in deep time.

Also, the long-chain n-alkane data needs to be included in the supplements too.

3) PAH data - petrogenic source?

PAHs are inferred to derive from biomass burning - but they can also form via thermal maturation (i.e. petrogenic PAHs) –

crucially, this can be assessed by comparing alkylated PAHs vs non-alkylated PAHs (see Karp or Lyons papers by Kate Freeman group, for example). I suggest using the alkylated PAH derivative index (APDI) to ensure that PAH signature is definitely from biomass burning.

Also, in the supplements, I noted that the F/F+P ratios range from 0.36 to 0.54 and average value is 0.40 suggesting petrogenic source – but the main text reports value of 0.61 (L145) suggesting biomass source - could the authors clarify please?

4) pH data?

If you have ombrotrophic bog, you should also have low pH conditions – the ratio of bb vs ab C31 hopanes has been used to infer pH in modern and ancient wetlands (see Inglis 2018, Inglis 2025, Lauretano 2021) – do you have hopanes in the hydrocarbon fraction? And if so, do you see dominance of C31 ab isomer > C31 bb isomer? If so, this could suggest low pH and support the presence of an acidic environment.

A cautionary note though – with increasing thermal maturation, you will naturally see a shift towards ab isomers – so high C31 ab isomers could be explained by that....

Minor comments:

L51: define age of late Cret
Fig. 2 – can you add age to y-axis? Even if its just “late Cret” and “Eocene” box that would be useful. Also suggest labelleing each subgraph (a), (b), (c) etc so its easier to match to caption text
C/N ratio in Fig 2 seems to suggest aquatic when values less than 20 (i.e. blue colours) but most algae are closer to 7 – you do have two values that are very low (~2 and ~4) – are these suggesting aquatic incursions?
L111: yes but sphagnum can also produce bimodal alkane distributions too (see studies above)
L125: this probably deserves a reference
L157: ref needed
L212: when did sphagnum really become important in Cenozoic? It’s a big time window so be a bit more specific
L217: putative sphagnum biomarkers – higher also produce 23/25 n-alkanes (see Bush and McInerery 2013). Its the ratio of mid-vs-long chain that is more specific, rather than just abundance of C23+C25.

Gordon Inglis

Reviewer #2

(Remarks to the Author)
SCIENTIFIC REVIEW REPORT - Co-evolution of wildfires and early Sphagnum-peatlands near the Cretaceous South Pole

General Evaluation:

The manuscript presents a multidisciplinary study addressing the relationship between wildfires and the early development of Sphagnum-dominated peatlands in Antarctica during the Late Cretaceous (Turonian–Santonian). The work integrates multiple lines of evidence, including palynology, organic geochemistry, charcoal analysis, inertinite reflectance, and biomarker data, providing a robust framework for reconstructing fire-related palaeoecological processes in polar environments.

The study is scientifically relevant and innovative, as it documents:
a. Evidence of wildfire activity in temperate forests located near the South Pole during the Cretaceous.
b. A potential ecological interaction between fire disturbance and the early development of Sphagnum-dominated peatlands.
c. The oldest record of coal-forming Sphagnum peatlands in southern high latitudes.
The manuscript is well structured and methodologically sound, addressing a topic of high relevance for studies of paleoclimate, fire paleoecology, and carbon-cycle dynamics during greenhouse intervals.

Originality and Scientific Contribution:

The principal contribution of this study is the demonstration that:
a. Wildfires were an intrinsic component of Cretaceous polar ecosystems, even within humid environments.
b. There may have been a co-evolutionary relationship between fire disturbance and hydrosere succession leading to Sphagnum-dominated peatlands.
c. Modern polar peatland systems may have functional analogues in greenhouse ecosystems of the geological past.
Furthermore, the record described represents:
d. The southernmost evidence of Cretaceous wildfire activity (~900 km from the South Pole).
e. Potentially the oldest record of Sphagnum-dominated peatlands associated with lignite formation at high southern latitudes.
These aspects confer substantial scientific value and potential impact to the manuscript.

Methodological Quality:

The study employs several complementary paleoenvironmental proxies.
a. Palynological analysis
Palynological data allow the inference of vegetation changes from Nyssa–Taxodium swamp forests to Sphagnum-dominated peatland environments, indicating ecological succession within wetland systems.
b. Charcoal analysis
The combined analysis of microcharcoal (<125 µm) and macrocharcoal (>125 µm) enables the reconstruction of both regional and local fire activity, which represents a methodologically appropriate approach.
c. Inertinite reflectance
Inertinite reflectance measurements indicate combustion temperatures of approximately 370–400 °C, suggesting low-intensity surface fires, characteristic of humid ecosystems and peatland environments.
d. Organic geochemistry
Organic geochemical analyses, including (PAHs), n-alkanes, and TOC/TN ratios, consistently support the interpretation of a terrestrial depositional environment with progressive peatland development.

Methodological assessment:

The use of multiple independent proxies strengthens the interpretations and constitutes one of the main strengths of the study.

Strengths of the Manuscript:

Multiproxy approach
The integration of palynology, organic geochemistry, and charcoal analysis provides a robust reconstruction of paleoenvironmental dynamics.

Paleoclimatic significance
The study contributes important evidence for understanding:
a. Polar ecosystems during greenhouse climate intervals.
b. The relationship between wildfire activity and peatland formation.

Aspects Requiring Improvement:

Origin and verification of the fire signal:
The manuscript briefly mentions possible ignition sources, but this aspect remains underdeveloped. The discussion could be expanded based on the available evidence, considering factors such as: Lightning, Climatic seasonality, Volcanic activity, Biomass accumulation.
In Figure 3, important botanical elements of the study are presented; however, the most critical evidence is absent. It is essential to include scanning electron microscope (SEM) imagery demonstrating that the studied material corresponds to charcoal produced by combustion.
According to Scott (2010) in the paper “Charcoal recognition, taphonomy and uses in palaeoenvironmental analysis” (Palaeogeography, Palaeoclimatology, Palaeoecology), the diagnostic characteristic of charcoal is the structural alteration produced during carbonization.
During carbonization, cell walls become homogenized, lose the original wood texture, and become optically more uniform. This transformation results from pyrolysis of plant material during wildfire events.
Therefore, studies demonstrating the occurrence of wildfires, regardless of geological age, should include SEM images showing homogenized cellular walls, which constitute a diagnostic feature of charcoal. This is fundamental!

Figurs:

Figure 1 - The clarity of the figure should be improved. It is currently difficult to identify the precise locations where previous studies have reported wildfire occurrences in Antarctica.
I recommend addressing this by:

a. Adding numbered markers for each locality with documented wildfire evidence.
b. Including the corresponding author and year in the figure legend.
c. Numbering the fire symbols shown in the figure.
Additionally, it is important to ensure that all currently known Antarctic wildfire records are represented in the figure. Several important references appear to be missing. Notably, the figure does not include the study reporting the first record of Cretaceous wildfire in Antarctica (Manfroi et al., 2015). This reference is present in the bibliography but absent from the figure, as are several others. Please revise the figure accordingly.

Figure 5- I understand that the authors opted to use AI-generated imagery to illustrate the paleoenvironmental reconstruction. However, given the significance of this study, a reconstruction produced by a professional paleoartist might provide a more scientifically grounded visualization. Nevertheless, the authors retain full autonomy regarding this decision, and the current image is generally consistent with the results presented in the study.

Minor Comments and Editorial Details:

Line 52: It would be appropriate to mention the intense volcanic activity at the end of the Cretaceous as an additional factor potentially associated with wildfire occurrence.

Editorial suggestions:
a. Correct minor typographical errors:
“charocal” (p. 8)
“predominantely” (p. 7)
b. Standardize the terminology used throughout the manuscript:
“peat bog”
“peatland”

Conclusion of the Review:

The manuscript represents a significant scientific contribution to the study of fire paleoecology and the evolution of peatland systems under greenhouse climate conditions.
The evidence presented suggests that:
a. Wildfires were part of Cretaceous polar peat-forming ecosystems dominated by Sphagnum.
b. This study documents the southernmost evidence low-temperature wildfires.

Recommendation
The manuscript is suitable for publication after minor but necessary revisions, particularly regarding:
a. Improvements to Figure 1.
b. The inclusion of evidence confirming the identification of charcoal (SEM).

Final Evaluation
High scientific quality and a strong contribution to the understanding of fire paleoecology and peatland evolution.
The study also provides valuable insights into the paleoenvironmental history of Antarctica.

Reviewer #3

(Remarks to the Author)
This paper reworks earlier results on the core and adds additional information not provided in the original publication(s). the authors document the presence of wildfire using a range of approaches that together give a well supported argument. However, wildfire in high latitudes is not new and indeed the authors reference various papers through Early and Late Cretaceous high southern latitude deposits that document wildfire in the landscape. there are however a few issues with parts of the text. In particular the identification of the wood. The authors report fenestriform cross field pitting but this is not illustrated and so therefore not verified. Figure 3e illustrates radial pitting of the tracheids but not crossfield pits and figure 3f while showing low magnification views of the ray cells also lacks details of this feature. Consequently the taxonomic assertions (line 200 to 204) in the text are not supported.

Line 51 late should read Late (and elsewhere e.g., line 52, 54, 57, 66, 83, 99, …… and throughout)
Line 66 not sure what is meant by West Antarctic mainland
Line 71 missing a “with” between along and biomarker
Line 96 (and elsewhere) no need to say families prior to Podocarpaceae the “aceae” ending indicates family.
Line 98 since should be replaced with during
Line 127 and 128 refers to wood anatomical features that are not supported by the illustrations provided. The so called cross field pits are not visible in Fig. 3e rather numerous pits in the radial walls on the tracheids are illustrated. The rays in figure 3f do not have any detail of cross field pits either.
Line 146 refers to burning of grass but convincing evidence of this group is near the K-T boundary somewhat younger than these deposits. There are earlier records but these are not considered reliable.
Line 200 to 204. The lack of convincing evidence for fenestriform cross field pitting makes these conclusions unsupported. Either provide evidence for the pit type or remove.

Version 1:

Reviewer comments:

Reviewer #2

(Remarks to the Author)
Following the review of the revised version of the manuscript entitled “Coevolution of Wildfires and Primitive Sphagnum Peatlands Near the Cretaceous South Pole”, I consider that the authors have adequately addressed the comments raised during the review process, incorporating the necessary modifications and clarifications to strengthen the scientific quality of the work. The manuscript presents novel and highly relevant results, providing significant evidence for understanding the interaction between wildfires and the early development of Sphagnum-dominated ecosystems in polar environments during the Cretaceous. Furthermore, it represents a valuable contribution to our knowledge of wildfire dynamics at high latitudes

and the paleoecological processes that characterized ecosystems near the South Pole during this period. Given the robustness of the data presented, the quality of the interpretations, and the importance of its scientific implications, I consider that the manuscript is suitable for publication.

I appreciate the opportunity to review this interesting study and congratulate the authors on their contribution to advancing knowledge in paleobotany, paleoecology, and wildfire research.

Kind regards.
Dra. Joseline Manfroi

Reviewer #3

(Remarks to the Author)
Firstly I thank the authors for their attention to the comments and detailed response to the questions raised.The comments in the initial review have been fully addressed and I have nothing further to add.

Open Access This Peer Review File is licensed under a Creative Commons Attribution 4.0 International License, which permits use, sharing, adaptation, distribution and reproduction in any medium or format, as long as you give appropriate credit to the original author(s) and the source, provide a link to the Creative Commons license, and indicate if changes were made.
In cases where reviewers are anonymous, credit should be given to 'Anonymous Referee' and the source.
The images or other third party material in this Peer Review File are included in the article's Creative Commons license, unless indicated otherwise in a credit line to the material. If material is not included in the article's Creative Commons license and your intended use is not permitted by statutory regulation or exceeds the permitted use, you will need to obtain permission directly from the copyright holder.
To view a copy of this license, visit https://creativecommons.org/licenses/by/4.0/

Response to Rev#1 Gordon Inglis for manuscript COMMSENV-26-0727-T

We thank **Gordon Inglis** for his very constructive comments and suggestions for improving our manuscript. First, we respond to the general comments before we address the more specific comments. We marked reviewer comments in italics and our respective response in green. Line numbers refer to the track change version.

Reviewer #1 (Remarks to the Author):

This is a well-written manuscript that provides the oldest known occurrence of a "modern-type" Sphagnum-dominated wetland in the geological record (as far as I am aware!). This pushes the oldest known occurrence back by 35 million years and thus will be of interest to the (paleo)ecological and (paleo)botanical community. The team use a wide variety of analysis to reach their conclusions - I do have a few comments about the biomarker interpretation but this does not affect the overall interpretation.
I have a couple of areas that I would like the authors to explore in more detail - but otherwise, I think this is a strong manuscript that would be well suited to this journal and be of interest to the Mesozoic and Cenozoic climate community.

1) Broader introduction
The introduction was concise and well written – but there are two areas that the authors may wish to discuss further.
First, it is worth noting the oldest occurrence of a "modern" Sphagnum-type bog in the geological record. By explaining that these records are scarce and rarely found beyond the Eocene (~55 Ma), it makes your conclusions more impactful. Second, perhaps the evolutionary aspects need to be discussed - when did Sphagnum evolve in the geological record? This paper (https://www.sciencedirect.com/science/article/pii/S1055790310000229#fig1) provides a nice overview of radiation of southern and northern hemisphere taxa

The oldest occurrence and evolutionary context of *Sphagnum* moss were briefly addressed in the Discussion section of the original submission (first paragraph of "Sphagnum-peat and changing fire regimes"), where we also cited the above-mentioned paper by Shaw et al. (2010). However, we agree with Rev#1 that the evolutionary framework should be introduced earlier to highlight the novelty of our findings. Accordingly, we have moved this material from the Discussion to the close of the Introduction. We have also expanded the relevant passage in the Discussion to note that the Cretaceous occurrence of "modern-type" *Sphagnum* at this locality raises questions about the prevailing assumption that Southern Hemisphere taxa derive from a late radiation from boreal-subarctic ancestors.

2) n-alkane data
It is interesting that there is an increase in C23 and C25 n-alkanes (putative sphagnum biomarkers) alongside an increase in sphagnum pollen – but what about other long-chain n-alkanes? Do they also increase? If they all increase, it might just suggest more plant input, rather than a shift in the type of plants.
The C23/C31 and C23/C29 n-alkane ratio has been proposed as a tracer for Sphagnum input into ancient peat deposits (see Nott 2000, Bingham 2010 and Bush and McInerey 2013). A number of studies have shown the close correspondence between these ratios and the relative abundance of Sphagnum leaves in a variety of modern bogs, but rarely applied in past – this ratio should therefore be added and the authors should evaluate whether this remains a useful chemotaxonomic tool in Cretaceous samples. When we applied this approach in the Schoningen early Eocene lignite, you see a strong increase in C23/C31 ratio from ~1 to ~7 alongside an increase in the sphagnum spores.
If your ratio does not change, that is fine - you clearly have loads of sphagnum pollen and I would not doubt the overall conclusions - but its useful for biomarker community to know how trustworthy this ratio is in deep time.
Also, the long-chain n-alkane data needs to be included in the supplements too.

We agree and have added this important point to the discussion. As the other long-chain n-alkanes also increase, the change might indeed be driven by the overall higher plant input during the succession from a swamp to a peat bog. This interpretation is further corroborated by elevated TOC/TN values (Fig. 2).

Following Rev#1's suggestion, we have now included the complete n-alkane data in the Supplementary Tables. We also added the C_{23}/C_{31} and C_{23}/C_{29} *n*-alkane ratios, which have previously been proposed as a *Sphagnum* tracer to the Supplementary Tables. In our record, these ratios do not appear to reliably reflect the pronounced *Sphagnum* maximum indicated by the spore assemblage. We therefore infer that the expansion of higher land plants, contributing predominantly long-chain n-alkanes, likely overprinted the *Sphagnum*-specific C_{23} and C_{25} signals. However, we agree with Rev#1 that this nonetheless represents valuable information for the biomarker community, and have therefore expanded the discussion, accordingly (see Lines 288-296)

3) PAH data - petrogenic source?

PAHs are inferred to derive from biomass burning - but they can also form via thermal maturation (i.e. petrogenic PAHs) – crucially, this can be assessed by comparing alkylated PAHs vs non-alkylated PAHs (see Karp or Lyons papers by Kate Freeman group, for example). I suggest using the alkylated PAH derivative index (APDI) to ensure that PAH signature is definitely from biomass burning.
Also, in the supplements, I noted that the F/F+P ratios range from 0.36 to 0.54 and average value is 0.40 suggesting petrogenic source – but the main text reports value of 0.61 (L145) suggesting biomass source - could the authors clarify please?

We thank Rev#1 for identifying this error. Following his suggestion, we have replaced the F/F+P ratios with the ADPI index to determine the PAH source. The revised analysis indicates a pyrogenic origin for the majority of samples (mid and lower depths), while the index reveals an increasing petrogenic influence towards the top of Core 9R. We interpret this shift as an overprint from the uppermost lignite layer. Biomass burning nevertheless remains evident from the abundant micro- and macro-charcoal, readily identifiable as inertinite/charcoal (see also new SEM image, Fig. 3d).

4) pH data?

If you have ombrotrophic bog, you should also have low pH conditions – the ratio of bb vs ab C31 hopanes has been used to infer pH in modern and ancient wetlands (see Inglis 2018, Inglis 2025, Lauretano 2021) – do you have hopanes in the hydrocarbon fraction? And if so, do you see dominance of C31 ab isomer > C31 bb isomer? If so, this could suggest low pH and support the presence of an acidic environment.
A cautionary note though – with increasing thermal maturation, you will naturally see a shift towards ab isomers – so high C31 ab isomers could be explained by that....

Following Rev#1's suggestion, we applied the C31 hopane $\beta\beta/(\alpha\beta + \beta\beta)$ index (Inglis et al., 2018) to selected samples from the mid to lower depths of core 10R and the uppermost part of core 9R to reconstruct pH values (see Supplementary Information). We also measured the C31 22S/(22R + 22S) ratios, which indicate low thermal maturity across all samples. The index points to an acidic environment for the uppermost peat samples, with pH values averaging ~4.3, compared to ~5 for the lowermost samples. These pH values are characteristic of ombrotrophic peat bogs.

Minor comments:

L51: define age of late Cret
Done

Fig. 2 – can you add age to y-axis? Even if its just "late Cret" and "Eocene" box that would be useful. Also suggest labelling each subgraph (a), (b), (c) etc so its easier to match to caption text
We added the ages on the right side of Fig. 2 and labelled each subgraph.

C/N ratio in Fig 2 seems to suggest aquatic when values less than 20 (i.e. blue colours) but most algae are closer to 7 – you do have two values that are very low (~2 and ~4) – are these suggesting aquatic incursions?

Both low measurements were obtained from the Eocene coastal sandstone, overlying the Cretaceous lignite layer. To avoid confusion, the uppermost Eocene low measurements for both n-alkanes and TOC/TN have been removed from Fig. 2 and the Supplementary Tables, as they are not discussed further and are not relevant to the findings of this paper.

L111: yes but sphagnum can also produce bimodal alkane distributions too (see studies above)
We expanded the discussion regarding alkane distributions. Please see reply to major comments above.

L125: this probably deserves a reference
Reference Crawford & Belcher (2022) added.

L157: ref needed
Reference Karp et al. (2020) added.

L212: when did sphagnum really become important in Cenozoic? It's a big time window so be a bit more specific
The timeline has been specified in the following sentences (Miocene expansion and Quaternary establishment). The entire paragraph has been moved to the end of the Introduction chapter.

L217: putative sphagnum biomarkers – higher also produce 23/25 n-alkanes (see Bush and McInerery 2013). Its the ratio of mid-vs-long chain that is more specific, rather than just abundance of C23+C25.
We agree and expanded the discussion regarding alkane distributions. Please see reply to major comments above.

Gordon Inglis

Response to Rev#2 s for manuscript COMMSENV-26-0727-T

We thank **Rev#2** for their positive and supporting general evaluation and constructive suggestions for improving our manuscript. Our response to "Aspects Requiring Improvement" can be found below. We marked reviewer comments in italics and our respective response in green. Line numbers refer to the track change version.

Reviewer #2 (Remarks to the Author):
General Evaluation:
The manuscript presents a multidisciplinary study addressing the relationship between wildfires and the early development of Sphagnum-dominated peatlands in Antarctica during the Late Cretaceous (Turonian–Santonian). The work integrates multiple lines of evidence, including palynology, organic geochemistry, charcoal analysis, inertinite reflectance, and biomarker data, providing a robust framework for reconstructing fire-related palaeoecological processes in polar environments.
The study is scientifically relevant and innovative, as it documents:
a. Evidence of wildfire activity in temperate forests located near the South Pole during the Cretaceous.
b. A potential …..

Originality and Scientific Contribution:
The principal contribution of this study is the demonstration that:
a. Wildfires were an intrinsic component of Cretaceous polar ecosystems, even within humid environments.
b. There may have been a co-evolutionary relationship between fire disturbance and hydrosere succession leading to Sphagnum-dominated peatlands.
c. Modern …….

Methodological Quality:
The study employs several complementary paleoenvironmental proxies.
a. Palynological analysis
Palynological data all…:

Strengths of the Manuscript:
Multiproxy approach
The integration of palynology….

Aspects Requiring Improvement:
Origin and verification of the fire signal:
The manuscript briefly mentions possible ignition sources, but this aspect remains underdeveloped. The discussion could be expanded based on the available evidence, considering factors such as: Lightning, Climatic seasonality, Volcanic activity, Biomass accumulation.

Following Rev#2's suggestion, we have expanded our discussion about potential ignition sources. We discuss potential volcanic activity, lighting, and seasonality (Lines 218-232).

In Figure 3, important botanical elements of the study are presented; however, the most critical evidence is absent. It is essential to include scanning electron microscope (SEM) imagery demonstrating that the studied material corresponds to charcoal produced by combustion. According to Scott (2010) in the paper "Charcoal recognition, taphonomy and uses in palaeoenvironmental analysis" (Palaeogeography, Palaeoclimatology, Palaeoecology), the diagnostic characteristic of charcoal is the structural alteration produced during carbonization.
During carbonization, cell walls become homogenized, lose the original wood texture, and become optically more uniform. This transformation results from pyrolysis of plant material during wildfire events.
Therefore, studies demonstrating the occurrence of wildfires, regardless of geological age, should include SEM images showing homogenized cellular walls, which constitute a diagnostic feature of charcoal. This is fundamental!

We thank Rev#2 for this suggestion and agree that homogenised cell walls, as described in Scott (2010), may represent a more definitive indicator of charred wood than the images previously shown in panels (d) and (f), which illustrated the brittle, blocky fracture pattern

and silky black lustre characteristic of charcoal. We have therefore removed these images and replaced them with an SEM image clearly demonstrating the presence of homogenised cell walls in our specimen.

Figures:
Figure 1 - The clarity of the figure should be improved. It is currently difficult to identify the precise locations where previous studies have reported wildfire occurrences in Antarctica.
I recommend addressing this by:
a. Adding numbered markers for each locality with documented wildfire evidence.
b. Including the corresponding author and year in the figure legend.
c. Numbering the fire symbols shown in the figure.
Additionally, it is important to ensure that all currently known Antarctic wildfire records are represented in the figure.
Several important references appear to be missing. Notably, the figure does not include the study reporting the first record of Cretaceous wildfire in Antarctica (Manfroi et al., 2015). This reference is present in the bibliography but absent from the figure, as are several others. Please revise the figure accordingly.

All references are included in both the text and figure. We acknowledge, however, that their exact positions may have been unclear, as sites were merged into groups with multiple references assigned per location. To address Rev#2's concern and minimise potential confusion, we have replaced the map in Fig. 1 with a more detailed version and have provided precise locations and corresponding references for each site in both the figure and the main text.

Please note that we are required to follow the journal's prescribed referencing style and therefore cannot provide full author names and publication years in the figure legend.

Figure 5- I understand that the authors opted to use AI-generated imagery to illustrate the paleoenvironmental reconstruction. However, given the significance of this study, a reconstruction produced by a professional paleoartist might provide a more scientifically grounded visualization. Nevertheless, the authors retain full autonomy regarding this decision, and the current image is generally consistent with the results presented in the study.

We are grateful for this suggestion and have welcomed palaeoartist James McKay (University of Leeds) to our author team. James previously painted the landscape reconstruction for our 2020 publication in *Nature*, and we are confident that his new illustration in Fig. 5 represents a substantial improvement over the AI-generated version. The reconstruction now more faithfully reflects a Southern Hemisphere vegetation character, evidenced by the palynological results.

Minor Comments and Editorial Details:
Line 52: It would be appropriate to mention the intense volcanic activity at the end of the Cretaceous as an additional factor potentially associated with wildfire occurrence.

The discussion of volcanic activity and other potential ignition sources has been expanded (see also response to preceding comment).

Editorial suggestions:
a. Correct minor typographical errors:
“charocal” (p. 8)
“predominantely” (p. 7)
Done

b. Standardize the terminology used throughout the manuscript:
“peat bog”

“peatland”

We have double checked the terminology and use throughout the text. “Peat bog” refers to the potentially ombrotrophic *Sphagnum* peat bog, whereas “Peatland” describes the wider wetland habitat which can include peat bogs, blanket peats and fens.

Conclusion of the Review:
The manuscript represents a significant scientific contribution to the study of fire paleoecology and the evolution of peatland systems under greenhouse climate conditions.
The evidence presented suggests that:
a. Wildfires were part of Cretaceous polar peat-forming ecosystems dominated by Sphagnum.
b. This study documents the southernmost evidence low-temperature wildfires.
Recommendation
The manuscript is suitable for publication after minor but necessary revisions, particularly regarding:
a. Improvements to Figure 1.
b. The inclusion of evidence confirming the identification of charcoal (SEM).
Final Evaluation
High scientific quality and a strong contribution to the understanding of fire paleoecology and peatland evolution.
The study also provides valuable insights into the paleoenvironmental history of Antarctica.

Response to Rev#3 for manuscript COMMSENV-26-0727-T

We thank **REV#3** for their constructive comments and suggestions for improving our manuscript. We marked reviewer comments in italics and our respective response in green.

Reviewer #3 (Remarks to the Author):
This paper reworks earlier results on the core and adds additional information not provided in the original publication(s). the authors document the presence of wildfire using a range of approaches that together give a well supported argument. However, wildfire in high latitudes is not new and indeed the authors reference various papers through Early and Late Cretaceous high southern latitude deposits that document wildfire in the landscape. there are however a few issues with parts of the text. In particular the identification of the wood. The authors report fenestriform cross field pitting but this is not illustrated and so therefore not verified. Figure 3e illustrates radial pitting of the tracheids but not crossfield pits and figure 3f while showing low magnification views of the ray cells also lacks details of this feature. Consequently the taxonomic assertions (line 200 to 204) in the text are not supported.

We thank Rev#2 for their overall supportive evaluation of our manuscript. However, we disagree with their concerns regarding our identification of the fossil wood material. To better support our interpretation, we have enlarged Figure 3e and added arrows to clearly indicate the location of the cross-field pits. We have also included, for direct comparison, a radial section image of modern *Lagarostrobos franklinii* wood, which demonstrates close morphological and anatomical similarities with the fossil specimen. In conifers, the pits on radial walls are uniformly sized within a given species. By contrast, the window-like or fenestriform pits, particularly those of the genera *Lagarostrobos*, *Manoao* and *Phyllocladus* exhibit considerable size variation, which is a feature already recognised in the early literature where they were described as 'egg pores' (Gothan, 1905). This variability should now be clearly visible in Figures 3e and the newly added Figure 3f.

We agree with Rev#2 that Figure 3f did not adequately illustrate the relevant morphological features. This image was originally included to show the characteristic black lustre of charcoal, rather than cross-field pit anatomy, and we acknowledge that its presence may have caused confusion. We have therefore removed it from the revised manuscript.

Regarding Fig. 3e, we would like to clarify that the availability of larger pieces of fossil charcoal is very limited as it was recovered from a (small) sediment core. The charcoal was split using a blade rather than sectioned, as cutting is not feasible: the material would crumble or be destroyed under conventional sectioning. This approach inevitably introduces slight deviations in the plane of exposure; however, we are confident that these do not affect the taxonomic identification.

We hope that the additional comparative material and the revised figure presentation satisfactorily address Rev#2's concerns, and that they are in agreement that the specimen has been correctly identified as Podocarpaceae. These findings are also supported by our palynological counts, which are dominated by pollen of Podocarpaceae (>50%).

Line 51 late should read Late (and elsewhere e.g., line 52, 54, 57, 66, 83, 99, …… and throughout)
Done.

Line 66 not sure what is meant by West Antarctic mainland
Changed to “West Antarctica”

Line 71 missing a “with” between along and biomarker
added

Line 96 (and elsewhere) no need to say families prior to Podocarpaceae the "aceae" ending indicates family.
deleted

Line 98 since should be replaced with during
agreed and replaced

Line 127 and 128 refers to wood anatomical features that are not supported by the illustrations provided. The so called cross field pits are not visible in Fig. 3e rather numerous pits in the radial walls on the tracheids are illustrated. The rays in figure 3f do not have any detail of cross field pits either.

As already mentioned above, Figure 3f was originally included to show the characteristic black lustre of charcoal, rather than cross-field pit anatomy, and we acknowledge that its presence may have caused confusion. We have therefore removed it from the revised manuscript. We hope that the revised, enlarged annotated image 3e, showing cross-field pits of different size in the radial wall, makes our identification more clearly understandable

Line 146 refers to burning of grass but convincing evidence of this group is near the K-T boundary somewhat younger than these deposits. There are earlier records but these are not considered reliable.

This referred to a methodological paper regarding PAH thresholds in general (and not for the Cretaceous). However, we removed the entire sentence to avoid confusion.

Line 200 to 204. The lack of convincing evidence for fenestriform cross field pitting makes these conclusions unsupported. Either provide evidence for the pit type or remove.

Please see response above.
