# Supplementary material for: Co-evolution of wildfires and early Sphagnum-peatlands near the Cretaceous South Pole
Source: Commun Earth Environ. 2026 Sep 7;7(1):715. doi: 10.1038/s43247-026-03959-1 (PMC13549950; doi:10.1038/s43247-026-03959-1)
Supplement: Supplementary file 2 [file 43247_2026_3959_MOESM2_ESM.docx]

**Description of Additional Supplementary files**

File name: Supplementary data Table 1

Description: Microscopic charcoal count

File name: Supplementary data Table 2

Description: Aspect ratio (length/width) of macroscopic charcoal particles (> 120 um)

File name: Supplementary data Table 3

Description: Palynology

File name: Supplementary data Table 4

Description: Polycyclic aromatic hydrocarbon analysis (PAH)

File name: Supplementary data Table 5

Description: Dimethyl phenanthrenes (DMP)

File name: Supplementary data Table 6

Description: Inertinite Reflectance

File name: Supplementary data Table 7

Description: n-alkanes

File name: Supplementary data Table 8

Description: Carbon to Nitrogen (TOC/TN) ratio

File name: Supplementary data Table 9

Description: Alkylated PAH derivative index (APDI)

File name: Supplementary data Table 10

Description: Hopanes
